# The relationship between work-family conflict and missed nursing care; a cross-sectional study in Iran

**DOI:** 10.1186/s12912-024-02556-x

**Published:** 2024-11-30

**Authors:** Mohammad Javad Ahmadzadeh-Zeidi, Zahra Rooddehghan, Shima Haghani

**Affiliations:** 1 Department Of Medical-Surgical Nursing, Faculty Of Nursing And Midwifery, Tehran University Of Medical Sciences, Tehran, Iran; 2 Department Of Biostatistics, Nursing And Midwifery Research Center, Faculty Of Nursing And Midwifery, Iran University Of Medical Sciences, Tehran, Iran

**Keywords:** Work-family conflict, Missed care, Nursing care, Iran

## Abstract

**Background & aims:**

Work–family conflict, an important issue in nursing management, has been examined extensively worldwide among nurses. Nurses’ inability to handle these conflicts affect their quality of care and may lead to missed nursing care. This study aimed to investigate the relationship between work-family conflict and missed nursing care in Iran.

**Materials & methods:**

This cross-sectional descriptive-analytical study was conducted on 270 nurses working in two hospitals affiliated to Tehran University of Medical Sciences (Sina & Shariati) from September to December 2023. The research community included all nurses working in medical-surgical wards in the designated hospitals. The research samples were included in the study in a targeted and quota sampling after obtaining organizational permits, ethical approval and informed consent. Data collection was done using three questionnaires including: Demographic information form, Carlson’s work-family conflict and Kalisch MISSCARE Questionnaire. The collected data was analyzed in SPSS software, version 25.

**Results:**

The results of Pearson’s correlation test showed that there is a positive relationship between nurses’ work-family conflict and missed nursing care scores (*r* = 0.21) (*p* = 0.001). The mean (SD) score of nurses’ work-family conflict was reported at a moderate level (61.58(11.57)) and the mean (SD) score of missed nursing care was reported at a low level (47.84(16.48)). Nurses under 29 years old and male nurses had more missed nursing care other than their participants. No more relationship was observed between other demographic variables with missed nursing care and nurses’ work-family conflict.

**Conclusion:**

According to the results of this research, it can be concluded that the existence of work-family conflict can be an obstacle for the correct implementation of nursing care. In such a way that nurses exposed to higher work-family conflict level had higher missed nursing care. Based on the findings of this research, it is recommended for health care providers and nursing managers to identify nurses exposed to high work-family conflicts, adopt programs to decrease their conflicts and consequently reduce missed nursing care.

## Background

In today’s societies, people are faced with various work and family responsibilities. The reason for the emergence of such responsibilities is the changes made in the nature of work and family [1, 2]. In some cases, the needs and requirements of the work and family environment are not aligned with each other, and sometimes employees lose the ability to establish effective connection between these two areas. Sometimes work issues interfere in the family and in some cases, family problems prevent doing work properly [3].

Work-family conflict is a two-way concept that includes both work-family interference and family-work interference [4]. The two-way nature of the conflict means that the pressures related to the roles can occur simultaneously from both directions and lead to a disturbance in another role [5]. Furthermore, Greenhouse and Beutel proposed different sources of conflict. Three other dimensions were also detected in each direction. These dimensions are time-based, Strain-based, and behavior-based, which ultimately lead to a six-dimensional structure for work-family conflict [6].

In the nursing profession, work-family conflict issues are common due to work pressures [7], lack of organizational support, low work flexibility [8], back-to-back shifts [9] and variables such as family responsibilities [10]. Studies in nursing show that work-family conflict is related to depressive symptoms, job and life dissatisfaction, emotional exhaustion [9], occupational hazards, decreased safety behavior [8] and decreased job performance [11].

According to the report of the International Labor Organization in 2019, one out of every three employees mentions the conflict between the work and family environment as one of the biggest problems in life [12]. Conflict between work and family is also inevitable in the nursing staff, because employees in this discipline experience a high level of physical, cognitive and emotional expectations [7] and this interference has been identified as one of the top 10 workplace stressors in the nursing profession [13].

In recent study, it has been shown that 50% of nurses experience chronic work interference with family and 11% of nurses experience chronic family interference at work [7]. The effects of excessive work and lack of proper balance between work and personal put the health of individuals and societies at serious risk [8]. Besides, the prevalence of conflict between healthcare workers with their occupational issues ranked fourth among all careers. In addition, the previous studies showed that the occupational stress has the highest ranked issue among healthcare workers [9]. In total, increasing the level of work-family conflicts has significantly reduced nurses’ job performance. On the other hand, with the increase in the level of psychological pressure in the work environment and the lack of time to complete the assigned tasks, in some situations nurses are not able to perform all their cares and prefer to prioritize some important cares, which results in doing some cares and forgetting some others [10].

Care is the central concept that distinguishes nursing from other health professions and it’s defined as the essence of nursing and complementary to the four meta-paradigmatic concepts of nursing [11]. One of the rights of hospitalized patients is to ensure that they receive safe and comprehensive care from the health care system. But in some situations, some care activities are being missed [12].

Missed Nursing Care was firstly introduced by Kalisch in 2006 [13]. Missed nursing care has recently been identified as any element of essential patient care that is partially or completely omitted by a nurse [14] and it is not only a type of nursing error, but it can also lead to ignoring patients’ rights and endangering their rehabilitation and recovery. since nurses can only handle a number of responsibilities in one shift, therefore, they tend to prioritize cares, and based on that decide which care to omit or which care to prioritize, which leads to forgetting some cares [12].

According to some studies, factors such as the inexperience of some nurses [15], long-term involvement with patients [16], overcrowding in the wards, Unfair division of responsibilities, excessive activities related to admission and discharge of patients [17] and “it’s not my job” syndrome have been mentioned in missed nursing care [18]. Some of the most common types of missed nursing care from the nurses’ viewpoint include: lack of oral care, failure to administer medicine at the appropriate time, failure to pay attention to the patient’s religious needs [19], delay in feeding and missed patient education and their discharge planning [16].

Depending on the research environment, financial and human resources around the world and Iran, The rate of missed nursing care varies between 10 and 50% (from low to high) which results in adverse complications for patients [20–22]. Among these, decrease in patient satisfaction, re-hospitalization, occurrence of medication errors [23] and increase in pain and discomfort of patients can be mentioned [16].

Comprehensive and high quality of care and patient safety is the ultimate goal of health care systems around the world [24]. the phenomenon of missed nursing care is still a serious threat to achieving comprehensive and safe nursing care, which also threatens the lives of patients [25]. Identifying the type of missed nursing care allows for effective strategies that can help maintain a healthy continuum of care [26].

In Iran, due to the lack of nursing staff and unfavorable economic conditions, nurses are working more than their duty hours, and then they are exposed to many conflicts between work and family due to acceptance various work and family responsibilities. The overflow of such conflicts in the work environment with a serious decrease in nurses’ job performance can significantly affect the main rights of patients to receive proper care. Also, failure to meet patients’ care needs may have various negative results depending on the type of missed care in each society. for this reasons, the relationship between work-family conflict and missed nursing care was investigated in Iran.

## Methods

### Design and aim

This is a cross-sectional and descriptive-analytical study, which was conducted with the aim of determining the relationship between work-family conflict and missed nursing care among nurses working in selected hospitals affiliated to Tehran University of Medical Sciences. This study was carried out from September to December 2023.

### Study setting and participants

In the current study, the research community included all nurses working in medical-surgical wards of selected hospitals affiliated to Tehran University of Medical Sciences, including: Sina and Dr. Shariati Hospital. These two hospitals were chosen purposefully as the research environment due to their alignment with the aim of this study as well as laws and guidelines they use. It should be noted that each of these hospitals has 16–18 medical-surgical wards and nurses from various regions of Iran are busy working there.

The inclusion criteria were; willing to participate in the study, being a nurse with a bachelor’s degree or higher education level, working in the medical-surgical wards, and having at least six months of clinical work experience, mental and physical health and the absence of stressful events such as divorce and death of relatives in the last six months. Since the working characteristics of general (medical-surgical) wards are different from special wards (ccu, icu, dialysis and emergency) in Iran, that is why the researchers decided to conduct this research in general wards. Exclusion criteria included incomplete answers to the questionnaire.

### Sample size and power

To determine the sample size at the confidence level of 95% and the test power of 90%, assuming that the correlation coefficient between work-family conflict and missed nursing care is at least 0.2 [2, 27], so that the relationship between the two variables is considered statistically significant, the sample size calculated 265 according to following formula:

$$\eqalign{& n = {{{{\left( {{z_{1 - {\alpha  \over 2}}} + {z_{1 - \beta }}} \right)}^2}} \over {{w^2}}} + 3,\,\,{\rm{w}}  \cr & \quad  = {1 \over 2}ln{{1 + r} \over {1 - r}}{\rm{,}}\,{\rm{w}} = {1 \over 2}ln{{1 + 0/2} \over {1 - 0/2}}  \cr & \quad  = 0/2,\,\,n = {{{{\left( {1/96 + 1/28} \right)}^2}} \over {0/{2^2}}} + 3 = 265 \cr} $$

A total of 270 nursing staff, including nurses with bachelor’s degree, master’s degree working as clinical nurses was selected by targeted and quota method. In the first step, after identifying these two hospitals, the quota of each hospital was determined according to the statistical population of nurses working in the medical-surgical wards. After that, according to nurses’ statistical population of each ward, the researcher started sampling nurses in three shifts: morning, evening and night. (168 nurses in DR. Shariati Hospital and 102 nurses in Sina Hospital).

### Instruments

Before conducting the research, permission was obtained from both creators of the localized version of these two questionnaires, and then the researchers began to conduct this study. The data collection in this study was based on the self-reporting method via three following questionnaires:

### Demographic information questionnaire

was used to collect information such as age, gender, marital status, employment status, work experience, education level, type of shift, and number of work shifts per month.

### Carlson’s work-family conflict questionnaire

This questionnaire was designed in 2000 by Carlson et al. This questionnaire has two main dimensions of work-family interference (e.g., “My work keeps me from my family activities more than I would like”) and family-work interference (e.g., “Due to stress at home, I am often preoccupied with family matters at work”) [28]. Each of these two main dimensions has its own time-based, strain-based, and behavior-based dimensions, which ultimately lead to a six-dimensional structure for work-family conflict [6]. The answer to each item of this 18-item questionnaire is on a 5-point Likert scale from completely disagree (1 point) to completely agree (5 points). In this way, the points obtained are between 18 and 90, where 18 points indicate the least conflict and 90 points indicate the most conflict. This questionnaire is without reverse score questions [28]. The validity of the work-family conflict questionnaire in Iran was firstly measured by Dargahi et al. and its reliability was obtained 0.84 using Cronbach’s alpha coefficient [8]. The reliability of this questionnaire was calculated as 0.89 using Cronbach’s alpha coefficient during this research.

**MISSCARE Questionnaire**: which was designed by Kalisch in 2006 to determine the items of missed nursing care and was psychometrically evaluated by the same researcher in 2009 [29]. Part A of the MISSCARE Questionnaire was used in this research and deals with the factors related to missed nursing care, which includes 24 nursing activities that are usually performed in each nursing shift [30]. This questionnaire has four subscales, which includes Assessment (8 items: e.g., Monitoring intake/output, Vital signs assessed as ordered), Interventions-Individual needs (6 items: e.g., assess effectiveness of medications, response to call light is provided within 5 min), Interventions-Basic Care (7 items: e.g., feeding patient when the food is still warm, patient bathing/skin care) and planning (3 items: e.g., Patient teaching, ensuring discharge planning). The answer to each item of this questionnaire is on a 5-point Likert scale including: never missed (score 1) to always missed (score 5) [23]. Scores of this questionnaire varies between 24 and 120, and a higher score indicates higher missed care [17]. The Persian translation and psychometric properties of this questionnaire was first done by Hosseini et al. and its’ validity and internal consistency was measured 0.93 [31]. The reliability of this questionnaire was calculated as 0.94 using Cronbach’s alpha coefficient during this research.

### Data collection

After obtaining the approval of ethics committee and the necessary permits from the relevant officials of the Faculty of Nursing and Midwifery of Tehran University of Medical Sciences, the researcher introduced himself to the directorates of selected hospitals. Then at the appropriate place and time the researcher explained the study objectives to nurses and obtained a written consent from them. All three questionnaires were equally distributed among nurses in three work shifts (morning, evening and night) and the samples were given 3 days to collect the questionnaires so that people can complete the questionnaires when they feel free and relaxed.

### Statistical analysis

Data were analyzed in SPSS software version 25. First, the Kolmogorov-Smirnov test was used to determine the normality of the data. After the data distribution was measured normal, data evaluation was done using descriptive statistics such as frequency and percentage, mean and standard deviation as well as inferential statistics such as independent t-test, ANOVA and Pearson’s correlation coefficient. The significance level of the data was considered as *p* < 0.05.

### Ethical considerations

This study was conducted by obtaining the code of ethics (IR.TUMS.FNM.REC.1402.091) from the ethical committees of the Nursing & Midwifery Faculty of Tehran University of Medical Sciences. After obtaining the approval of ethics committee and the necessary permits from the relevant authorities, the researcher introduced himself to directorates of selected hospitals. A well-trained and experienced research assistant explained study objectives to nurses and told them that their participation was voluntary and refusal to participate would not result in any negative consequences. All participants were assured of the confidentiality of all the information of the research and only the researchers have access to the data. Then informed consent was obtained from all study participants. Finally, the results of the study were provided to the hospitals under study.

## Results

After distributing about 330 questionnaires, 270 participants who had completed all three questionnaires were participated in the study. Of these 270 participants, 215 (79.6%) were female, 156 (57.8%) were single. The mean (SD) age was 31.41(7.56) and the mean (SD) work experience (year) was 6.91(6.68). 238 (88.1%) had a bachelor’s degree and more than 220 (81.5%) had no children. Other demographic information are given in Table 1.

**Table 1:** Demographic characteristics of the nurses participating in the study

Variables		Number (%)
Gender	Male	55(20.4)
	female	215(79.6)
Marital status	Single	156(57.8)
	married	114(42.2)
Education level	Bachelor	238(88.1)
	Master	32(11.9)
Type of shift	Fix	104(38.5)
	Circular	166(61.5)
employment status	Yes (official nurses)	156(58)
	No (contract nurses)	114(42)
Age, Mean ± SD		31.41 ± 7.56
Number of shift per month, Mean ± SD		29 ± 4.28
Work experience (year), Mean ± SD		6.91 ± 6.68

The mean (SD) score of nurses’ work-family conflict was 61.85(11.57), which indicates moderate work-family conflict. The results showed that the highest score of the work-family conflict dimension is related to “time-based work-family interference” with a mean (SD) score of 12.59(2.57) (range 3–15) and the lowest score related to " Strain-based family-work interference” with a mean (SD) score of 8.51(3.27) (range 3–15). During this study, the mean score of work-family interference was found to be higher than family-work interference. (Table 2).

**Table 2:** Nurses’ work-family conflict and missed nursing care scores of the participants (*n* = 270)

Variables	The number of items per subscale	Mean (SD)	1–5 basis Mean (SD)
**Work-Family Conflict (scores: 18–90)**	18	61.85 (11.57)	3.44 (0.64)
Time-based work-family interference (3–15)	3	12.5(2.57)	4.20 (0.85)
Time-based family-work interference (3–15)	3	9.81 (2.69)	3.27 (0.89)
Strain-based work-family interference (3–15)	3	11.99 (2.69)	4.00 (0.89)
Strain-based family-work interference (3–15)	3	8.51 (3.27)	2.84 (1.09)
Behavior-based work-family interference (3–15)	3	9.54 (2.49)	3.18 (0.83)
Behavior-based family-work interference (3–15)	3	9.41 (2.72)	3.14 (0.90)
Work-family interference (9–45)	9	34.11 (6.11)	3.79 (0.67)
Family-work interference (9–45)	9	27.73 (6.85)	3.08 (0.76)
**Missed Nursing Care (scores: 24–120)**	24	47.84 (16.48)	1.99 (0.68)
Assessment (8–40)	8	13.74 (6.07)	1.72 (0.75)
Interventions-Individual needs (6–30)	6	12.22 (4.32)	2.04 (0.72)
Interventions-Basic Care (7–35)	7	15.27 (5.84)	2.18 (0.83)
Planning (3–15)	3	6.61 (2.45)	2.20 (0.81)

The mean (SD) score of missed nursing care was calculated as 47.84(16.48) (1.99(0.68) on 1–5 basis), which indicates that the missed care is at a low level. Also, “planning for nursing care” has the highest rate of missed care compared to other aspects of nursing care with a mean (SD) score of 2.20(0.81) and “patient assessment” with a mean (SD) score of 1.72(0.75) has the lowest rate of missed care based on score of 1–5 (Table 2). In this research, " Attend interdisciplinary care conferences whenever held” had the highest degree of missed care and “Bedside glucose monitoring as ordered” had the lowest degree of missed care.

Nurses under 29 years old and male nurses had more missed cares than other participants. No more relationship was observed between other demographic variables with missed nursing care and nurses’ work-family conflict (Table 3).

**Table 3:** The relationship between demographic characteristics of participants with work-family conflict and missed nursing care (*n* = 270)

		Work-Family Conflict		Missed Care	
demographic characteristics		**Mean (SD)**	**Test result**	**Mean (SD)**	**Test result**
Age^①^	≤ 29	61.53 (11.18)	*F = 0.26	50.91 (16.12)	***F = 496
	30–39	62.57 (10.29)		44.71 (15.77)	
	≥ 40	61.36 (15.17)		44.62 (17.59)	
Work experience^①^	< 5	61.72 (10.89)	*F = 2.09	49.25 (15.32)	*F = 1.77
	5–9	60.34 (11.74)		48.70 (18.91)	
	10–14	66.96 (11.93)		45.31 (15.72)	
	≥ 15	61.13 (13.08)		42.95 (17.01)	
Number of shift per month^①^	< 20	63.80 (9.64)	*F = 0.88	43.20 (13.52)	*F = 0.84
	20–24	59.06 (14.45)		45.23 (16.55)	
	25–29	62.40 (13.87)		48.24 (17.41)	
	≥ 30	62.08 (10.12)		47.84 (16.40)	
Gender^②^	Male	61.82 (11.39)	*t=-0.08 df = 268	53.76 (18.09)	***t = 3.03 df = 268
	Female	61.96 (12.37)		46.33 (15.73)	
Marital status^②^	Single	61.88 (10.85)	*t = 0.05 df = 268	48.83 (16.09)	*t = 1.14 df = 268
	Married	61.81 (12.45)		46.50 (16.67)	
Education level^②^	Bachelor	62.01 (11.90)	*t = 0.62 df = 268	48.01 (16.88)	*t = 0.45 df = 268
	Master	60.66 (8.85)		46.59 (13.26)	
employment status^②^	Yes (official)	61.83 (12.80)	*t = 1.25 df = 268	48.98 ± 17.17	*t = 0.86 df = 268
	No (contract)	60.72 (12.19)		49.54 ± 15.99	
Type of shift^①^	Fix morning	58.74 (14.20)	*F = 1.07	42.26 (15.17)	*F = 1.45
	Fix evening	64.18 (10.07)		51.09 (12.60)	
	Fix long	63.56 (8.09)		45.50 (16.95)	
	Fix night	60.48 (13.71)		50.39 (15.45)	
	Circular	61.98 (11.26)		48.10 (17.37)	

Note acceptable p value is *p* < 0.05. ① Analysis of variance (ANOVA) test and ② t-test for independent groups. *p > 0.05 **p < 0.05, ***p < 0.01

The results of the Pearson correlation test showed that there is a positive relationship between work-family conflict and missed nursing care (*p* < 0.001 and *r* = 0.21). Also, the most negative impact of work-family conflict was on the “planning” field in nursing care (*p* < 0.001 and *r* = 0.24) (Table 4).

**Table 4:** Pearson correlation between work-family conflict and missed nursing care

Variables		Pearson correlation	*P*-value
**Work-Family Conflict**	Assessment	*r* = 0.21	*p* < 0.001
	Interventions-Individual needs	*r* = 0.17	*p* = 0.005
	Interventions-Basic Care	*r* = 0.16	*p* = 0.008
	planning	*r* = 0.24	*p* < 0.001
	Missed Nursing Care	*r* = 0.21	*p* = 0.001
**Missed Nursing Care**	Time-based work-family interference	*r* = 0.11	*p* = 0.061
	Time-based family-work interference	*r* = 0.14	*p* = 0.015
	Strain-based work-family interference	*r* = 0.18	*p* = 0.002
	Strain-based family-work interference	*r* = 0.17	*p* = 0.003
	Behavior-based work-family interference	*r* = 0.13	*p* = 0.023
	Behavior-based family-work interference	*r* = 0.14	*p* = 0.015
	Work-family interference	*r* = 0.18	*p* = 0.002
	Family-work interference	*r* = 0.20	*p* = 0.001
	Work-family conflict	*r* = 0.21	*p* = 0.001

Note acceptable p value is *p* < 0.05

## Discussion

The present study was conducted in order to determine the relationship between work-family conflict and missed nursing care in the medical-surgical wards of selected hospitals affiliated to Tehran University of Medical Sciences.

According to the findings of this research, work-family conflict had a statistically weak relationship with missed nursing care. This state means that other factors besides work-family conflicts may have relationship with missed nursing care. For example, some studies in Iran have stated that missed nursing care is unavoidable due to systemic and managerial problems and lack of organizational support [14, 16]. In addition to these, back-to-back shifts, forced overtime, severe shortage of nursing personnel and lack of attention to the family needs by nursing managers have led to an increase in the level of work-family conflict among Iranian nurses. This issue has caused more missed nursing care in such nurses. Consistent with our finding in Iran [32] and Oman, as the level of work-family conflict increased, the quality of nursing care also decreased [33].

The most negative relationship of work-family conflict was related to “planning for nursing care”. In general, it can be said that, cares that are not recorded in any part of the nursing report sheet and are not checked by nurses during shift delivery, can be one of the important causes of forgetting such cares [12].

The results indicate moderate work-family conflict in Iranian nurses. Consistent with our findings the mean scores of nurses’ work-family conflict was reported at a moderate level in Turkey [34], China [35], Iran [36] While in other studies mean scores was reported at a high level [8, 37]. Consistent with our findings, scores of missed nursing care in Jordanian and Iranian nurses was reported at a below moderate level [21, 38]. While in another study, scores were reported at upper moderate [20, 39] and at a high level [22, 40, 41].

The difference in the scores of work-family conflict and missed care can be attributed to the difference in the workload of the personnel and the lack of workforce, in addition to the existence of cultural and organizational differences in the study areas. With increasing workloads coupled with ongoing nurse shortages, it is not surprising that many nurses are prioritizing clinical care over those activities that are not documented and deemed necessary [42].

In this study mean scores of work-family interference was reported higher than family-work interference. Because of being in unusual working conditions, insomnia and related problems, nurses tolerate more work-family interference, and for this reason, their work roles are in conflict with their parental roles [43].

The results of this research show that the highest score of the work-family conflict dimension was related to “time-based work-family interference”. In Iranian societies, people usually work more than the prescribed time per week to meet their living expenses. Increasing working hours and not having enough time to take care of family affairs may cause them to experience more conflict in terms of time [43].

In this regard, Greenhaus & Beutell stated that high involvement in a role causes conflicts between roles to increase in two ways: First, high involvement in a role can increase the time devoted to that role. As a result, it will be more difficult to match the expectations of the second role. Second, an increase in conflict in a role causes an increase in mental engagement with that role [44]. This can lead to a strong internal addiction to work hard that cannot be resisted. Therefore, such an uncontrollable process, which is the desire to focus intensely on work, causes interference in other family matters such as handling family responsibilities [45].

The findings of the research showed that the mean score of missed nursing care in Iranian nurses is at a low level. Also, “planning for nursing care” and “interventions-basic care” each have the highest rate of forgetting with 8% and “patient assessment” with 4% have the lowest rate of forgetting compared to other aspects of nursing care. The overall rate of missed nursing care during this study was 5%.

It seems that missed care such as attend interdisciplinary care conferences whenever held, assess effectiveness of medications, Response to call light and in general, cares that are not recorded in any part of the nursing report and are not checked by nurses during shift delivery, can be one of the causes of missing such cares. Also, according to attend interdisciplinary care conferences, which had the highest rate of forgetting, it can be said that the absence of a codified program and synchronization with nurses’ free time and the lack of importance of such conferences for nurses caused nurses to forget this part of care [12].

In this study “patient assessment” has the lowest rate of missed care. It can be said that nurses pay a lot of attention to this type of care due to recording these cares in the patient’s file and transferring this information during shift delivery. On the other hand, in our studied society, laws, regulations and guidelines have the first power from nurses’ viewpoint, and they consider themselves obliged to comply with them. That’s why cares that needs to be recorded and documented, such as the cares that are associated with the examination and assessment of the patient, are less neglected [21].

Also, the results showed that there is no relationship between demographic variables and work-family conflict, which is in line with the study of Mosalanezhad et al. [8]. While other studies indicate higher work-family conflict among female nurses. It seems that this can be caused by the simultaneous occupation and family roles of female nurses based on the cultural views in the society [2, 46]. Also, in some studies, with increasing age and work experience, the level of work-family conflict increased (47, 48). While in another study, this relationship was interpreted negatively (49).

Incompatibility of roles leads the working person to increase stress, depression at work and possibly, decrease the establishment of personal connections in the work and home environment. Researchers and experts have also mentioned, among the consequences of work-family conflict, the reduction of adherence to organizational ethics, resorting to behaviors that involve avoiding work responsibilities, disruption in effective communication and emotional breakdown with patients [2]. Also, nurses who cannot effectively communicate between their family and work demands, suffer from mental turmoil and not be able to spend enough energy and time to respond to their work and family needs. This situation has led to the degradation of the individual’s energy levels, which disrupts the vitality and energy of working at the workplace and causes a serious decrease in job performance. The first unfavorable effects of these conflicts in the work environment can be seen in the quality of patient care and reduced adherence to organizational responsibilities [46]. One of the consequences of this decreased job performance can be seen in the quality of nursing care and missed nursing care, which results in an increased risk of side effects for patients. In this regard, the results of a study showed that missed nursing care can lead to various outcomes such as nosocomial infections, falls, and medication errors, which ultimately increase prolonged hospitalizations, readmissions, and even death [19]. This is why the mutual effects of work-family conflict and missed nursing care can have a great impact on the health care systems.

### Strengths and limitations

Since 2009, many studies have been conducted on work-family conflict and its causes. However, there has been no study in Iran that wants to examine the relationship between work-family conflict and its’ dimensions on missed nursing care.

Also, this study was conducted on two hospitals (Sina and Dr. Shariati) which are located in the capital of Iran (Tehran) and nurses from various regions of Iran are busy working there. This maybe made research result more generalizable. The researcher tried to collect data equally in all three shifts, morning, evening and night, so that the results of the research are closer to reality.

### Research limitations include

the psychological states of the participants and using data collection method based on the participants’ self-report, was beyond the researcher’s control. This situation may lead to giving false information to the researchers. Also, despite ensuring the confidentiality of the information, the fear of revealing the information may cause the participants to respond unrealistically. Also, fatigue and lack of time caused by work-family conflict in nurses may have caused unfocused and wrong answers to questions. Moreover, this study used a cross-sectional design, which means the study results are limited to reflecting only those conditions experienced during the data collection process. The occurrence of a stressful events may seriously change the level of behavior-based and strain-based conflicts during the completion of the questionnaires, which ultimately, change the research results. Since the working characteristics of general (medical-surgical) wards are different from special wards (CCU^1^, ICU^2^, dialysis and emergency) in Iran, that is why the researchers decided to conduct this research in general wards. According to the researcher’s opinion, the research results in these two working conditions may be different. It is better to conduct another research with the same title in special wards.

## Conclusions

According to the results obtained from the present study, it can be concluded that the existence of work-family conflict can be an obstacle for the correct implementation of nursing care. So that nurses with a higher level of work-family conflict had higher missed nursing care. Time-based work-family interference was identified as the most important form of work-family conflict among Iranian nurses. “Planning for nursing care” has the highest rate of missed care and “patient assessment” has the lowest rate of missed care compared to other nursing care subscales.

Also, the ultimate goal of all health care systems is to increase the quality of care and increase patients’ satisfaction. For this reason, it is recommended for health care providers and nursing managers to identify nurses exposed to high work-family conflict, adopt programs to decrease their work-family conflict and consequently reduce missed nursing care.

## Applications of this study

### Application in nursing management

An increase in work-family conflict causes nurses’ work demotivation and increased missed nursing care. The occurrence of this situation can lead to a decrease in the overall efficiency of the organization and departure of more human resources from the organization. Also, by using the findings of this research, hospital nursing officials and managers can more accurately find the causes of work-family conflict and missed nursing care in each wards and adopt necessary plans to solve them.

### Application in nursing education

Acquainting nurses with the phenomenon of work-family conflict and the necessary training methods to reduce its effects on working conditions and reduce occupational and family tensions, can encourage them to provide better and higher quality nursing care.

There is also a need for health system policy makers to put a special educational course on their agenda in this field, which has received less attention.

### Application in clinical practice

Reducing work-family conflict through reducing stress and mental pressure in different wards, can increase interpersonal interactions and increased work efficiency, which results can be seen in reducing missed nursing care.

#### Suggestion for further studies

Since the working characteristics of general (medical-surgical) wards are different from special wards (CCU, ICU, Dialysis, Emergency) in Iran, that is why the researchers decided to conduct this research in general wards. According to the researchers’ opinion, the research results in these two working conditions may be different. It is better to conduct another research with the same topic in special wards.

This study suggests that studies should be conducted to measure the in-service education programs focusing on Conflict management methods in nurses and its impact on missed nursing care.

## Data Availability

The datasets are available from the corresponding author on reasonable request.

## Footnotes

1 Cardiac Care Unit.

2 Intensive Care Unit.
